# Supplementary material for: Facemasks: Perceptions and use in an ED population during COVID-19
Source: PLoS One. 2022 Apr 13;17(4):e0266148. doi: 10.1371/journal.pone.0266148 (PMC9007380; doi:10.1371/journal.pone.0266148)
Supplement: S1 File — (DOCX) [file pone.0266148.s001.docx]

**ED Based COVID-19 Prevention Program Survey**

1. Have you previously been diagnosed with COVID-19 or had a positive COVID-19 test?

- 1. Yes
  2. No
  3. Unsure

2. Do you wear a mask when you are outside of your home and you are around other people?

a. Always

b. Most of the time (more than 50%)

c. Sometimes, but less than half of the time (less than 50%)

d. I never wear a mask

2A. If you wear a mask, where did you get the mask? (check all that apply)

a. From a store or pharmacy

b. From the ER

c. From my doctor or clinic

d. From a shelter or food bank

e. I ordered it (them) online

f. A friend or family member gave it to me

g. Other_______________

2B. If you wear a mask, do you believe you have enough of them?

a. Yes, I have enough masks available to me (and my family)

b. No, I don’t have enough masks right now, but can easily get more

c. No, I don’t have enough masks and it is difficult for me to get more

2C. If you “never wear a mask”, list the reason(s) why not? (pick all that apply)

a. I don’t have a mask and it is hard for me to get one

b. I don’t believe that masks work

c. I was previously wearing masks but I ran out

d. Others have pressured me not to wear a mask

e. The masks make it hard to do my work

f. The masks are uncomfortable on my face

g. I was previously wearing masks but got tired of wearing them

h. The masks make it hard for me to breathe

i. Other________________

6. What is your age: ___

7. What is your gender? (check one)

Male

Female

Trans Male

Trans Female

Genderqueer/Gender Non- binary

Decline to answer

8. What is your race/ethnicity? (check all that apply)

African-American/Black

Asian

Hispanic/Latinx

Middle Eastern/Chaldean

Native American /American Indian

Native Hawaiian or Pacific Islander

White (non-Latinx)

Other________

Decline to answer

1. Do you currently have a place to live:
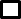
 Yes
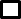
 No

9A. If NO, how long have you been homeless?

- - - < 1 month
    - 1-6 months
    - 6 months – 1 year
    - > 1 year

9B. Do you live with anyone?

- No, I live alone
- Yes

9C. If yes, how many people do live with? ____

9D. If you with people, which of the following live with you? (check all that apply)

Roommate(s)/friends

My spouse/partner(s)

Children under age of 18

Parent or Parents

Someone over the age of 60

Decline to answer

10. What is your primary language?

- - English
  - Spanish
  - Cantonese/Mandarin
  - Tagalog
  - Arabic
  - Bengali
  - Other: _______________________

10A**.** If English is not your primary language, how well do *you* speak and understand English?

- - Not at all
  - A little
  - Most of it
  - All of it (completely)

11**.** Do you have health insurance:
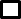
 Yes
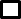
 No
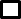
 I am currently applying for it
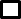
 Unsure

11A. If YES, what type?
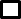
 Private
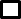
Medicare
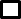
 MediCal
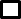
 Kaiser
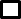
 Affordable Care Act (ObamaCare)
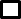
 Military of Veterans Administration
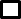
 Healthy San Francisco
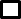
 Other

12**.** Do you have a regular clinic or doctor for medical care?
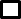
 Yes
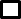
 No

12A. If YES, when was the last time you saw *this* doctor or went to the clinic?

- - - < 1 month
    - 1-6 months
    - 6 months – 1 year
    - > 1 year

12B. If NO, when was the last time you saw *any* doctor or had health care in the U.S. (besides today)?

- - - < 1 month
    - 1-6 months
    - 6 months – 1 year
    - > 1 year
    - I have never seen a doctor in the U.S.

12C. If NO to regular doctor, where do you usually go when you are sick or need medical advice?

- - - An emergency department
    - A clinic
    - Urgent care center
    - Other________________________
    - I don’t remember
