# Supplementary material for: Facemasks: Perceptions and use in an ED population during COVID-19
Source: PLoS One. 2022 Apr 13;17(4):e0266148. doi: 10.1371/journal.pone.0266148 (PMC9007380; doi:10.1371/journal.pone.0266148)
Supplement: S1 Table — (DOCX) [file pone.0266148.s002.docx]

**S1 Table: Demographic information by mask wearing frequency**

| **Do you wear a mask when you are outside your home or around other people?** | | | | |  |
| --- | --- | --- | --- | --- | --- |
|  | **Total** | **Never** | **Sometimes** | **Most of the Time** | **Always** |
| **n (%)** | 2,292 | 14 (0.6%) | 81 (3%) | 394 (17%) | 1803 (79%) |
| **Age (mean(SD))** | 48 (17) | 52 (18) | 45 (17) | 45 (17) | 49 (17) |
| **Gender, (n(%))** |  |  |  |  |  |
| Female | 1,129 | 7 (0.5%) | 21 (2%) | 159 (14%) | 942 (83%) |
| Male | 1,146 | 7 (0.6%) | 58 (5%) | 232 (20%) | 849 (74%) |
| Other | 8 | 0 (0%) | 1 (12%) | 1 (12%) | 6 (75%) |
| Missing | 9 | 0 (0%) | 1 (11%) | 2 (22%) | 6 (67%) |
| **Race, (n(%))** |  |  |  |  |  |
| Latinx | 535 | 2 | 13 | 91 | 429 |
| White, non-Latinx | 853 | 11 (1%) | 35 (4%) | 153 (18%) | 654 (77%) |
| Black, non-Latinx | 667 | 1 (0%) | 28 (4%) | 116 (17%) | 522 (78%) |
| Asian, non-Latinx | 94 | 0 (0%) | 1 (1%) | 9 (10%) | 84 (89%) |
| Middle Eastern, non-Latinx | 26 | 0 (0%) | 0 (0%) | 4 (15%) | 22 (85%) |
| Native American, non-Latinx | 12 | 0 (0%) | 3 (25%) | 0 (0%) | 9 (75%) |
| Native Hawaiian, non-Latinx | 6 | 0 (0%) | 0 (0%) | 2 (33%) | 4 (67%) |
| Other | 34 | 0 (0%) | 0 (0%) | 4 (12%) | 30 (88%) |
| Multiracial | 41 | 0 (0%) | 0 (0%) | 7 (17%) | 34 (83%) |
| Decline | 12 | 0 (0%) | 0 (0%) | 5 (42%) | 7 (58%) |
| Missing | 12 | 0 (0%) | 1 (8%) | 3 (25%) | 8 (67%) |
| **Homeless, (n(%))** |  |  |  |  |  |
| Yes | 84 | 2 (2%) | 14 (17%) | 17 (20%) | 51 (61%) |
| **Insured, (n(%))** |  |  |  |  |  |
| Yes | 1,998 | 14 (0.7%) | 68 (3%) | 320 (16%) | 1596 (80%) |
| No | 294 | 0 (0%) | 13 (4%) | 74 (25%) | 207 (70%) |
| **Insurance Type, (n(%))** |  |  |  |  |  |
| Private | 871 | 1 (0.1%) | 16 (2%) | 137 (16%) | 717 (82%) |
| VA | 13 | 0 (0%) | 1 (8%) | 6 (46%) | 6 (46%) |
| ACA | 99 | 2 (2%) | 11 (11%) | 8 (8%) | 78 (79%) |
| Medicare | 377 | 3 (0.8%) | 15 (4%) | 52 (14%) | 307 (81%) |
| Medicaid | 540 | 8 (1%) | 20 (4%) | 103 (19%) | 409 (76%) |
| Kaiser | 20 | 0 (0%) | 1 (5%) | 3 (15%) | 16 (80%) |
| Local Community Health Plan | 31 | 0 (0%) | 0 (0%) | 6 (19%) | 25 (81%) |
| Uninsured | 278 | 0 (0%) | 14 (5%) | 60 (22%) | 204 (73%) |
| Unsure | 45 | 0 (0%) | 1 (2%) | 18 (40%) | 26 (58%) |
| Missing | 18 | 0 (0%) | 2 (11%) | 1 (6%) | 15 (83%) |
| **Hospital Region, (n(%))** |  |  |  |  |  |
| North East | 457 | 2 (0.4%) | 12 (3%) | 81 (18%) | 362 (79%) |
| Midwest | 451 | 5 (1%) | 20 (4%) | 67 (15%) | 359 (80%) |
| South | 444 | 4 (0.9%) | 21 (5%) | 78 (18%) | 341 (77%) |
| West Coast | 939 | 3 (0.3%) | 28 (3%) | 167 (18%) | 741 (79%) |
| Missing | 1 | 0 (0%) | 0 (0%) | 1 (100%) | 0 (0%) |
| **Primary Language, (n(%))** |  |  |  |  |  |
| English | 1,848 | 13 (0.7%) | 74 (4%) | 325 (18%) | 1,436 (78%) |
| Spanish | 344 | 1 (0.3%) | 6 (2%) | 55 (16%) | 282 (82%) |
| Other | 95 | 0 (0%) | 0 (0%) | 13 (14%) | 82 (86%) |
| Missing | 5 | 0 (0%) | 1 (20%) | 1 (20%) | 3 (60%) |
| **Have a Primary Care Physician, (n(%))** |  |  |  |  |  |
| No | 423 | 4 (1%) | 28 (7%) | 81 (19%) | 310 (73%) |
| Yes | 1,859 | 10 (0.5%) | 53 (3%) | 312 (17%) | 1,484 (80%) |
| Missing | 10 | 0 (0%) | 0 (0%) | 1 (10%) | 9 (90%) |
| **Usual Source of Care, (n(%))** |  |  |  |  |  |
| Primary Care Physician | 1,859 | 10 (0.5%) | 53 (3%) | 312 (17%) | 1,484 (80%) |
| ED | 274 | 3 (1%) | 23 (8%) | 52 (19%) | 196 (72%) |
| Clinic | 60 | 0 (0%) | 1 (2%) | 17 (28%) | 42 (70%) |
| Urgent Care | 51 | 0 (0%) | 1 (2%) | 8 (16%) | 42 (82%) |
| Unsure | 25 | 0 (0%) | 1 (4%) | 3 (12%) | 21 (84%) |
| Other | 23 | 1 (4%) | 2 (9%) | 2 (9%) | 18 (78%) |
| **Previous COVID-19 Diagnosis, (n(%))** |  |  |  |  |  |
| No | 1,910 | 14 (1%) | 69 (4%) | 320 (17%) | 1507 (79%) |
| Yes | 341 | 0 (0%) | 19 (3%) | 63 (18%) | 268 (79%) |
| Unsure | 38 | 0 (0%) | 2 (5%) | 11 (29%) | 25 (66%) |
| Missing | 3 | 0 (0%) | 0 (0%) | 0 (0%) | 3 (100%) |
